# Effect of carrier confinement on effective mass of excitons and estimation of ultralow disorder in Al_*x*_Ga_1−*x*_As/GaAs quantum wells by magneto-photoluminescence

**DOI:** 10.1038/s41598-017-05139-w

**Published:** 2017-07-07

**Authors:** S. Haldar, V. K. Dixit, Geetanjali Vashisht, Shailesh Kumar Khamari, S. Porwal, T. K. Sharma, S. M. Oak

**Affiliations:** 1 Semiconductor Physics and Devices Laboratory, Solid State Laser Division, Raja Ramanna Center for Advanced Technology, Indore, Madhya Pradesh 452013 India; 2 Homi Bhabha National Institute, Anushakti Nagar, Mumbai, 400094 India

## Abstract

Effect of charge carrier confinement and ultra-low disorder acquainted in AlGaAs/GaAs multi-quantum well system is investigated via Magneto-photoluminescence spectroscopy. Significant increase of effective mass is observed for the confined exciton in narrow QWs. The foremost reason behind such an observation is due to the induced non-parabolicity in bands. Moreover, as the thickness of the QW are reduced, confined excitons in QW experience atomic irregularities at the hetero-junctions and their effects are prominent in the photoluminescence linewidth. Amount of photoluminescence line-broadening caused by the atomic irregularities at the hetero-junctions is correlated with average fluctuation (*δ*_1_) in QW thickness. The estimated *δ*_1_ for Al_0.3_Ga_0.7_As/GaAs QWs are found to be ±(0.14 − 1.6)× ‘one monolayer thickness of GaAs layer’. Further, the strong perturbations due to magnetic field in a system helps in realizing optical properties of exciton in QWs, where magnetic field is used as a probe to detect ultralow defects in the QW. Additionally, the influence of magnetic field on the free and bound exciton luminescence is explained by a simple model. The proposed approach for measuring the interface and volume defects in an ultra-low disordered system by Magneto-PL spectroscopy technique will be highly beneficial in high mobility devices for advanced applications.

## Introduction

Among the III–V compound semiconductor hetero-structures, Al_*x*_Ga_1−*x*_As/GaAs based hetero-junctions and quantum structures occupy a privileged position owing to very high crystalline quality epitaxial layer with minimal defects and ultralow lattice disorder^1–3^. These are some of the key properties of Al_*x*_Ga_1−*x*_As/GaAs hetero-junctions that are mainly responsible for the observation of extremely high mobility of charge carriers and enhanced efficiency of numerous opto-electronic devices^4–7^. Recent investigations on various innovative proof of concept demonstrations in arsenide based semiconductor hetero-structures, let it be induced superconductivity or spin-electronics, have considerably renewed the interest towards ultra-low disordered AlGaAs/GaAs heterojunction and quantum structures^8–10^. In order to design and fabricate advanced quantum structures, a quantitative estimate of disorders present in the epitaxial layers and also at the hetero-interface is mandatory. In addition to this, a simultaneous in-depth understanding on the scattering mechanisms (limits the mobility) of charge carrier, which is primarily governed by the quality of heterojunction and disorders present in the system, is essential^6^. In general, classical and quantum Hall effect experiments are carried out to understand the scattering mechanisms of charge carrier in semiconductor hetero-structures^9, 11^. However, formation of Ohmic contact in undoped and small dimensioned sample is often challenging. Moreover, the technique is destructive since the sample is no more useful after measurement. In view of this, contactless measurement techniques like photoluminescence (PL), surface photo voltage (SPV) spectroscopy are attractive to probe the surface and interface defects^12–14^. It has been qualitatively understood that the linewidth of PL signal is determined by the extent of inhomogeneities and defects of the QW^15–19^. Aksenov *et al*. and Oliveira *et al*. had pre-assumed that the fluctuation (*δ*_1_) in the width of QWs as one monolayer thickness, and subsequently they have estimated the lateral inhomogeneities at the hetero-junction of the QWs^17, 18^. Bansal *et al*.^16^ reported that the reduction in inhomogeneously broadened PL linewidth of AlGaAs/GaAs QWs at high magnetic field, which might be contributed by the interface disorder. However, no quantitative information on the disorders in QW was given by them. Further, the penetration of wave function into the barrier layer and its impact on the linewidth is not accounted in their analysis^16^. Irrespective of extensive investigation, both theoretically and experimentally, the complex behavior of PL linewidth is not properly understood yet. In addition, it is extremely important to investigate the effect of wave function penetration into the barrier layer on the optical properties by varying the thickness of the QW layer. However, one need to keep the sample parameters like intentional/unintentional dopant density, disorder and defect density constant. It can be achieved by growing a multi-quantum wells (MQWs) sample in a single growth run. Effective mass (m*) of carriers is a crucial parameter, which primarily governs the limit to the charge carrier mobility. However, one cannot unambiguously determine the effective mass of carriers in a MQW sample by using electronic transport measurements due to the problems associated with parallel layer conduction. In view of this, contactless spectroscopic measurements like PL and surface photo-voltage (SPV) in presence of high magnetic field (B) is really attractive, where it is possible to decouple the accurate values of m* for all the QWs in a MQW sample.

With these in mind, we have performed systematic Magneto-PL experiments on a MQW sample. The non-parabolicity in bands are found to be the dominant factors in determining the values of effective mass (m*) for a thin QW. It is also noticed that the simple picture of parabolic band is not sufficient to explain the higher value of reduced effective mass of QWs. One need to consider the effect of non-parabolicity in bands and penetration of wave function into the barrier layer to understand why a large value of the effective mass is obtained. Further, the atomic irregularities at the hetero-junction, resulting fluctuation in QW thickness (*δ*_1_) is quantitatively estimated by modeling the PL linewidth. Subsequently, effect of point defects distributed in x-y plane (volume defects) on PL line-shape is also investigated from Magneto-PL spectroscopy. A decrease in the asymmetry of PL spectra and enhancement of PL intensity are observed at high magnetic field. A simple model, considering the magnetic field driven in-plane confinement of exciton, is constructed to explain such effects in AlGaAs/GaAs MQWs.

## Methods

AlGaAs/GaAs MQW structure is grown using metal organic vapor phase epitaxy (MOVPE) technique on (001) GaAs substrate. Four GaAs QWs with thickness 190, 100, 50 and 30 Å are periodically sandwiched with 940 Å thick Al_0.3_Ga_0.7_As barrier layers, which are labeled as QW-1, QW-2, QW-3 and QW-4 respectively. These structural parameters are estimated by matching high-resolution x-ray diffraction (HRXRD) pattern with simulated results through X’Pert epitaxy software. Thicknesses of the QWs are further confirmed by matching the experimental and theoretical values of transition energies measured from the PL spectrum. Magneto-PL experiments are carried out by Janis research cryostat (SVT-2513-DMI) experimental setup, where the MQW sample is kept in liquid helium bath (T = 4.2 K), inside a Dewar of the thermostat. In order to perform Magneto-PL experiment in high magnetic field up to 8 T (perpendicular to sample surface, *B*_*z*_), MQW sample is surrounded with helical shaped niobium-titanium (NbTi) superconducting magnet. Frequency doubled Nd:YAG green laser (*λ* = 532 nm) is used for the non-resonant excitation of AlGaAs/GaAs MQW sample. Excitation light is transported through an optical fiber where the typical spot size is about 500 *μ*m in diameter. Same optical fiber is used to collect the PL signal. In order to minimize the intensity dependent effects, such as saturation of energy levels, linewidth broadening, temperature rise effects etc., the power density of the excitation source on the surface of the sample is limited to only 0.51 W/cm^2^ with the help of neutral density filter. Generally, these intensity dependent effects can be observed considerably for the power density greater than 10–12 W/cm^2^ ^20^. The Magneto-PL spectrum is dispersed by monochromator and detected by Si photodiode using lock-in amplifier technique.

## Results and Discussion

**Figure 1:**
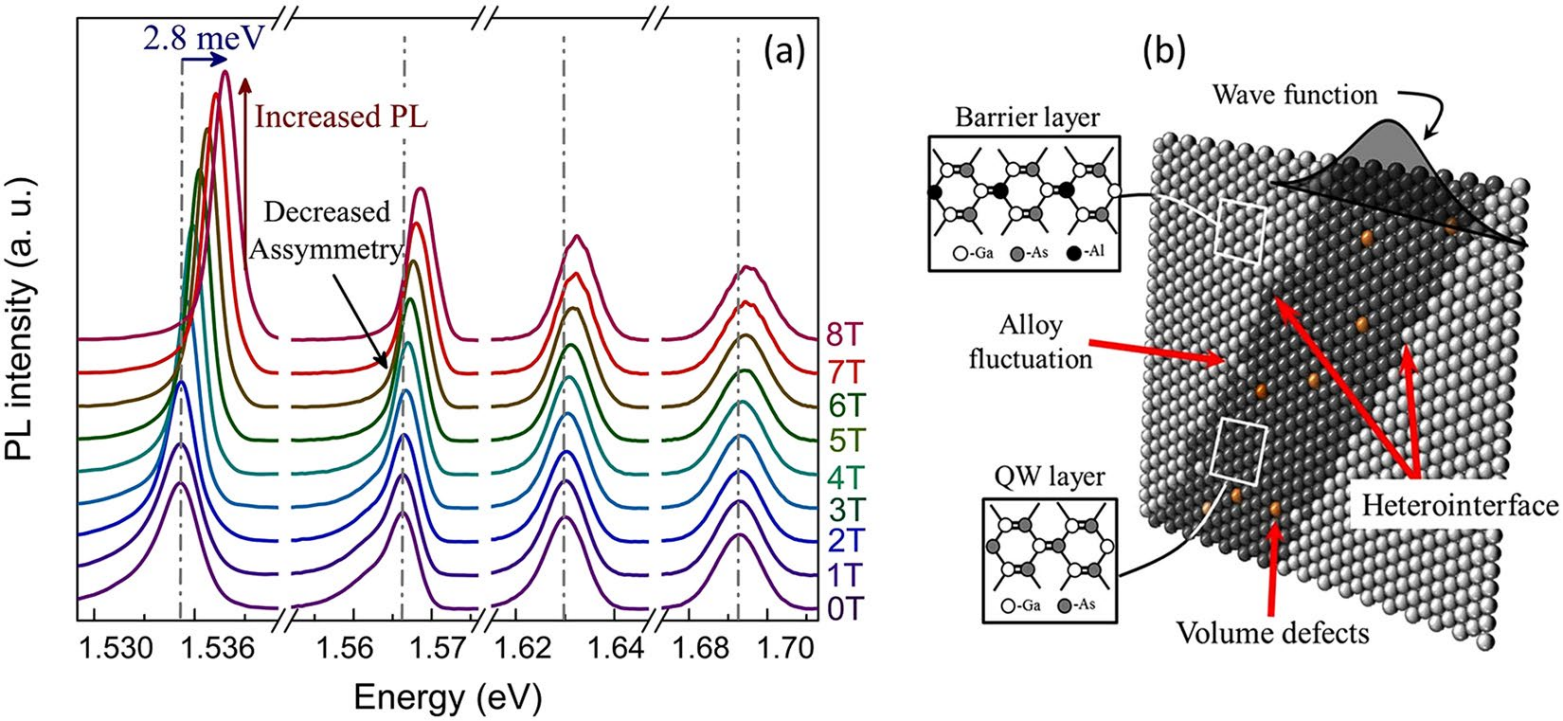
(**a**) PL Spectrum of AlGaAs/GaAs MQW sample at 4.2 K. Broadening in linewidth is more pronounced in thin QWs. Magnetic field dependent blue shift in PL line-shape is also observed. Asymmetry in PL line-shape is predominantly observed in thick QWs (≥50 Å). In presence of magnetic field, PL intensity increases and asymmetry in line-shape reduces. (**b**) Schematic cross-sectional view of AlGaAs/GaAs QW displaying hetero-interface atomic irregularities, volume defects, compositional and thickness fluctuation and the penetration of wave function into the barrier layer.

Figure 1(a) shows the Magneto-PL spectra of AlGaAs/GaAs MQW sample recorded as a function of magnetic field at 4.2 K. PL line-shape at *B* = 0 T seems to be predominantly asymmetrically broadened for all the QWs. Additionally, the PL intensity and asymmetry in PL line-shape increase with increase in QW width (*l*). A monotonic rise in PL linewidth with decrease in QW thickness [Fig. 1(a)] indicates that the carriers of narrow QW experience added interfacial-inhomogeneity and undergoes the strong influence of barrier layer. PL linewidth is primarily governed by several factors such as, 1) natural broadening i.e. homogeneous broadening (${\rm{\Delta }}{E}_{Natural}$), 2) defect/disorder in well layer i.e. point defects distributed in x-y plane, (${\rm{\Delta }}{E}_{Volume}$), 3) potential fluctuation caused by the compositional fluctuation into the barrier layer (${\rm{\Delta }}{E}_{Pot\mathrm{.}fluct\mathrm{.}}$), 4) thickness fluctuation of the QW layer (${\rm{\Delta }}{E}_{Thickness}$), and 5) penetration of exciton wave function into the barrier layer (${\rm{\Delta }}{E}_{penetration}$), as shown in Fig. 1(b). Scattering of charge carriers/excitons by the defects in the system may result in the distribution of energy of excitons in energy space, which may lead to the broadening of PL linewidth. The energy broadening of PL spectra originating from various scattering sources can be described as follows^21–24^,

1 $${\rm{\Delta }}{E}_{Total}^{2}={\rm{\Delta }}{E}_{Natural}^{2}+{\rm{\Delta }}{E}_{Volume}^{2}+{\rm{\Delta }}{E}_{Pot\mathrm{.}fluct\mathrm{.}}^{2}+{\rm{\Delta }}{E}_{Thickness}^{2}+{\rm{\Delta }}{E}_{penetration}^{2}$$

**Figure 2:**
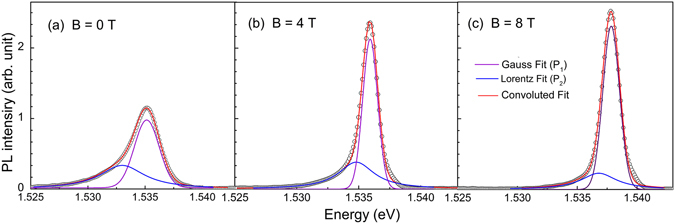
4.2 K PL spectra (open circles) of 190 Å thick QW (QW-1) in MQW sample at (**a**) 0, (**b**) 4 and (**c**) 8 T magnetic field. PL spectra is deconvoluted by Gaussian components *P*_1_ (purple curve) and Lorentz component *P*_2_ (blue curve) to fit the asymmetric PL line shape (red curve).

The strain induced contribution to inhomogeneous broadening is neglected in our case due to small lattice mismatch between GaAs and AlGaAs crystals. Further, AlGaAs/GaAs QWs possess ultra-low disorder in it, and highly uniform electronic charge environment in lattice. Therefore, the effect of spectral diffusion is also not considered in our Magneto-PL line-shape analysis. It is to be noted that the disorders in a system may significantly influence the dynamics of charge carrier and also influence various radiative and non-radiative mechanisms in a QW. After uniform generation of photo-excited carrier in the sample, charge carriers diffuse to the minimum potential area originated by volume defects, QW thickness fluctuation (due to the hetero-junctional atomic irregularity), compositional irregularity, etc. The PL line-shape is very sensitive to the density and energy distribution of surface and interface states. Total PL signal can be expressed by the rate equation of energy levels considering the radiative/non radiative lifetime of various states that are associated in a transition. It has been shown previously that the migration lifetime and radiative recombination lifetime of charge carrier in a QW play a crucial role in determining the line-shape of PL spectrum^25^. Magneto-PL spectra [Fig. 1(a)] show that the asymmetry in PL line-shape of all the QWs seem to disappear at higher magnetic field (8 T). Therefore, in order to understand the effect of magnetic field on the line-shape of PL and its energy, PL line-shapes are fitted with Gaussian along with Lorentz function^18, 26^, with desired accuracy (goodness of fit, *R* ^2^ ≥ 0.995) as shown in Fig. 2. Gaussian function peak *P*_1_, which shows relatively large integrated PL intensity for all the QWs in MQW sample is understood to be originating from the radiative recombination of free excitons. Inhomogeneously broadened Gaussian function peak *P*_1_ is influenced by a set of scattering mechanisms such as natural broadening, compositional fluctuation, atomic irregularity at the heterojunction, penetration of wave function into the barrier layer, etc^27^. On the other hand, homogeneously broadened Lorenz peak *P*_2_, is mainly originated from shallow defect/disorder and impurity bound exciton^21, 28^. Further, in the PL spectra of our undoped AlGaAs/GaAs MQW sample, distinct features related to donor/acceptor bound exciton or trion have not been observed^29^. However, the defect or the impurity bound exciton *P*_2_ may have slight donor type nature, due to the presence of Si^30^. Magnetic field dependent diamagnetic blue shift of PL spectra (*P*_1_) is proportional to the square of *B* (B < B_*c*_)^31, 32^,

2 $${\rm{\Delta }}E(B)={R}_{D}\frac{{e}^{2}{B}^{2}{r}_{B}^{2}}{4{\mu }^{\ast }}=\alpha {R}_{D}{B}^{2}$$

However, at relatively higher magnetic field, excitons are confined to very small radius (at *B* = 8 T radius *r* ≈ 90.7 Å)^31^. This magnetic field driven in plane confinement of exciton is responsible for the formation of discrete Landau levels^33^. In this high field regime (B ≥ B_*c*_), blue shift in energy levels become proportional to the applied field *B*.

3 $${\rm{\Delta }}E(B)=\frac{\hslash eB}{2{\mu }^{\ast }}=\beta B$$

**Figure 3:**
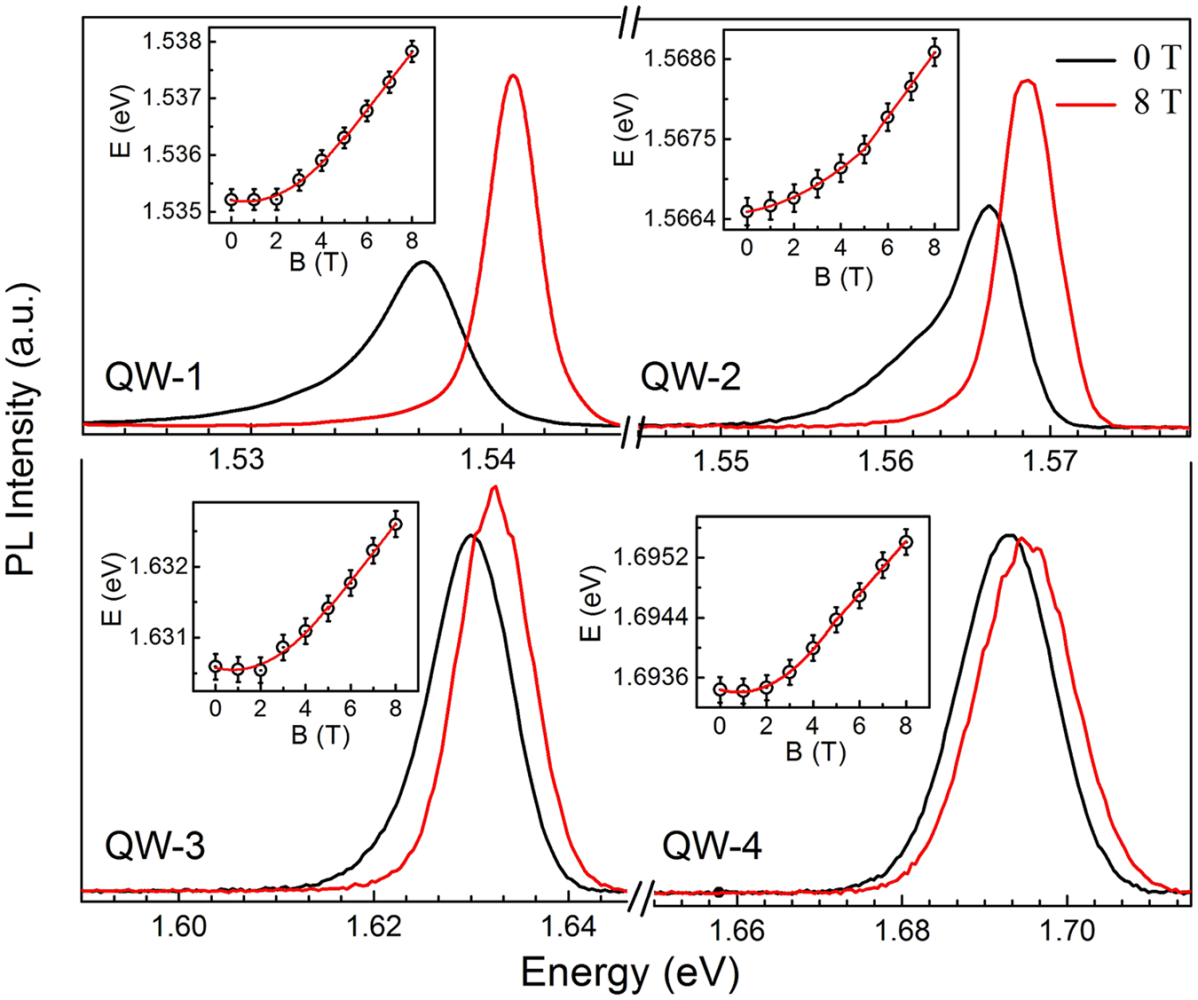
4.2 K PL spectra of QW-1 (190 Å), QW-2 (100 Å), QW-3 (50 Å), QW-4 (30 Å) at 0 T (black curve) and 8 T (red curve) magnetic field. Blue shift of PL peak energy (ΔE) as a function of *B* is shown in the respective insets.

Here, *R*_*D*_ and *r*_*B*_ are the dimensionality factor and Bohr radius respectively, and *α*, *β* are the proportionality constants. μ* is the reduced effective mass of exciton and can be expressed as, $1/{\mu }^{\ast }=1/{m}_{e}^{\ast }+1/{m}_{h}^{\ast }$, with ${m}_{e}^{\ast }$ and ${m}_{h}^{\ast }$ are the effective mass of electron and hole, respectively. The magnetic field, above which magnetic energy becomes dominant over Columbic energy, is termed as critical magnetic field (${B}_{c}={\mu }^{\mathrm{\ast 2}}{e}^{3}/16{\pi }^{2}{\varepsilon }_{0}^{2}{\varepsilon }_{r}^{2}{\hslash }^{3}$)^31^, and is theoretically estimated as 4.9 T for GaAs. Here, *ε*_*r*_ = 13.18 is the dielectric constant of GaAs^34^, *ε*_0_ is the permittivity of free space and *ħ* is the (Planck’s constant)/2*π*. Thereafter, Eq. 3 is used to estimate the reduced effective mass of excitons for the four QWs using the Magneto-PL spectra at high filed regime *B* ≥ 5 T (Fig. 3)^32, 35, 36^. Excitons are the bound state of two oppositely charged particles, i.e. electron and hole and stable under the force of centrifugal and Coulombic attraction. A greater confinement of charge carrier in the QWs results in significant amount of wave function penetration into the barrier layer. Therefore, the zero field binding energy of excitons may be approximated by ${E}_{b}(B=\mathrm{0)}\approx ({\mu }^{\ast }{e}^{4}/32{\pi }^{2}{\varepsilon }_{0}^{2}{\varepsilon }_{r}^{2}{\hslash }^{2})$ ^31^, and can be computed by the experimentally estimated reduced effective mass of excitons. The estimated exciton binding energy for the four QWs are summarized in Table 1, which is close to the previously reported values estimated by more rigorous and elaborated theoretical and experimental calculation^37–39^.

**Table 1:** Summary of reduced effective mass and binding energy of exciton with variation in QW thickness (*m*_0_ is the free electron mass).

*l* (Å)	μ* ± Error (×*m*_0_)	*E*_*b*_ ± Error (meV)
190	0.114 ± 0.002	8.9 ± 0.4
100	0.133 ± 0.002	10.4 ± 0.6
50	0.163 ± 0.006	12.7 ± 1.0
30	0.178 ± 0.007	13.9 ± 1.1

### Effect of non-parabolicity in conduction band

The estimated reduced effective mass of excitons for all the QWs (Table 1) are higher than the reduced effective mass of either the GaAs well layer [μ*(GaAs) = 0.056 *m*_0_]^40^ or the barrier [μ*(Al_0.3_Ga_0.7_As) = 0.081 *m*_0_]^34^. One plausible explanation for the increase of effective mass with decrease in QW thickness may be the effect of wave function penetration into the barrier layer. As the dimension of a QW is reduced, penetration of wave function upsurges into the barrier layer, with simultaneous increase in reduced effective mass of exciton. Total effective mass (hole/electron) due to the fractional wave function penetration into the barrier layer can be estimated from the relation given below,

4 $$\frac{1}{{m}^{\ast }(QW+penetration)}=\frac{f}{{m}_{Well}^{\ast }}+\frac{(1-f)}{{m}_{Barrier}^{\ast }}$$

where, *f* and (*1* − *f* ) represents the occupation probability of electron/hole inside the well and barrier layer respectively. ${m}_{Well}^{\ast }$ and ${m}_{Barrier}^{\ast }$ stand for the effective mass of electrons/holes corresponding to GaAs QW layer and AlGaAs barrier layer respectively. Therefore, the increase in effective mass due to wave function penetration into the barrier layer can be expressed as,

5 $${\rm{\Delta }}{m}_{penetration}^{\ast }=\frac{{m}_{Well}^{\ast }(1-f)({m}_{Barrier}^{\ast }-{m}_{Well}^{\ast })}{f\times {m}_{Barrier}^{\ast }+\mathrm{(1}-f)\times {m}_{Well}^{\ast }}$$

**Figure 4:**
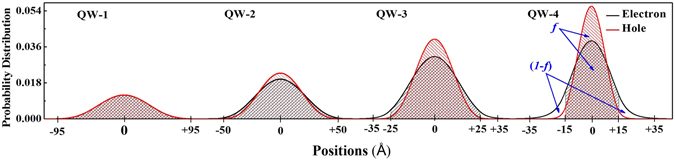
Spatial extent of electron/hole wave function computed by solving Schrödinger equation in FDM technique. Probability of finding an electron/hole in QW layer (*f*) and barrier layer (*1* − *f*) are estimated numerically.

The values of *f* for the electrons/holes are estimated by solving Schrödinger equation in finite difference method (FDM, Fig. 4), and subsequently ${\rm{\Delta }}{m}_{penetration}$ is estimated (Table 2). The consideration of effective mass due to the QW layer and wave function penetration into the barrier layer is not sufficient to justify the experimentally observed higher values of exciton reduced effective mass (Table 2). Therefore, the major contribution of this increased value of exciton reduced effective mass that are confined in QWs, could be due to the significant effect of non-parabolicity of bands. Several groups have theoretically studied the effect of non-parabolicity (NP) in conduction band in explaining this higher value of μ* for confined excitons in QWs^41–44^. According to those models, complete Hamiltonian of confined charge carriers in a QW in presence of magnetic field can be expressed as follows^45–47^,

6 $$\begin{array}{rcl}H & = & \frac{{\hslash }^{2}{k}^{2}}{2{\mu }^{\ast }}+\frac{1}{2}g{\mu }_{B}\sigma B+{\rm{\Gamma }}\sigma \varphi +{a}_{1}{k}^{4}+\frac{{a}_{2}}{{\xi }_{B}^{4}}\\  &  & +{a}_{3}[\{{k}_{x}^{2},{k}_{y}^{2}\}+\{{k}_{x}^{2},{k}_{z}^{2}\}+\{{k}_{y}^{2},{k}_{z}^{2}\}]\\  &  & +{a}_{4}{k}^{2}\sigma B+{a}_{5}(\sigma k,kB)+{a}_{6}{k}^{2}B\sigma +V(z)\end{array}$$

Here, g is the Lande g-factor, ${\rm{\Gamma }}$ is the Dresselhaus spin orbit coupling constant, *ϕ* is a vector operator with components ${\varphi }_{x}=({k}_{y}{k}_{x}{k}_{y}-{k}_{z}{k}_{x}{k}_{z})$, ${\xi }_{B}=\sqrt{\hslash /eB}$ is the magnetic length, and *σ* is the Pauli spin matrices. Lande g-factor for GaAs crystal is very small (−0.44 at 5 K)^45^, which results in negligible Zeeman energy induced into the system (0.2 meV at 8 T). Further, GaAs have negligible inversion symmetry, therefore, the third term of the Hamiltonian, in representing spin-orbit interaction, does not contribute in our case^45^. Term with *B* ^2^ dependency (containing *a*_2_) denotes the diamagnetic shift of energy levels due to the magnetic field. Terms containing *a*_1_ and *a*_3_ jointly contribute to the non-parabolicity in bands with *k* ^4^ dependency in dispersion relation. *V*(*z*) denotes the potential due to the barrier layer that is experienced by the confined excitons in QWs. Details of equation 6 may be found somewhere else^45, 46^. On the other hand, Hiroshima and Lang proposed that the non-parabolicity in conduction band can affect the effective mass of charge carrier, and under the non-parabolic consideration dispersion relation of band gets modified as^42^,

7 $${E}_{e}^{^{\prime} }=\frac{{\hslash }^{2}}{2{m}_{e}^{\ast }}{k}_{e}^{^{\prime} 2}(1-\gamma {k}_{e}^{^{\prime} 2})$$

where, (${E}_{e}^{^{\prime} }$, ${k}_{e}^{^{\prime} }$) represents energy and wavenumber corresponding to the non-parabolic conduction band, and may not be same as parabolic state (*E*_*e*_, *k*_*e*_). The symbol *γ* represents the non-parabolicity factor for the conduction band. Equation 7 is actually a simplified form of equation 6, which is a good approximation for the crystals like GaAs under above conditions. It is to be noted that at small wavenumber (${k}_{e}^{^{\prime} }\to 0$) i.e. close to band edge, equation 7 turns out to be the parabolic dispersion relation ${E}_{e}={\hslash }^{2}{k}_{e}^{2}/2{m}_{e}^{\ast }$, where ${m}_{e}^{\ast }$ (=${d}^{2}{E}_{e}/d{k}_{e}^{2}$ = 0.067 *m*_0_)^34^ is the mass of electron at the band edge. Therefore, the second order derivative of non-parabolic dispersion relation [Eq. 7] can be expressed as follows,

8 $${[\frac{{\partial }^{2}{E}_{e}^{^{\prime} }}{\partial {k}_{e}^{^{\prime} 2}}]}_{{E}_{e1}^{^{\prime} }}=\frac{{\hslash }^{2}}{{m}_{e}(expt\mathrm{.)}}=\frac{{\hslash }^{2}}{{m}_{e}^{\ast }}-\frac{6\gamma {\hslash }^{2}{k}_{e1}^{^{\prime} 2}}{{m}_{e}^{\ast }}$$

Here, [${\hslash }^{2}/({d}^{2}{E}_{e}^{^{\prime} }/d{k}_{e}^{^{\prime} 2})$] signifies the energy dependent effective mass of electrons at the non-parabolic conduction band, which is same as the experimentally estimated effective mass of electron [*m*_*e*_(*expt*.)]. (${E}_{e1}^{^{\prime} }$, ${k}_{e1}^{^{\prime} }$) represents the ground state of electrons at the non-parabolic conduction band of a QW. Using equation 7, above relation (eq. 8) can be further expressed to,

9 $$\gamma =[\frac{{m}_{e}(expt\mathrm{.)}-{m}_{e}^{\ast }}{6{m}_{e}(expt\mathrm{.)}{k}_{e1}^{^{\prime} 2}}]=\frac{[{m}_{e}(expt\mathrm{.)}-{m}_{e}^{\ast }]\times \mathrm{[5}{m}_{e}(expt\mathrm{.)}+{m}_{e}^{\ast }]}{72{m}_{e}^{\ast }{[{m}_{e}(expt\mathrm{.)]}}^{2}{E}_{e1}\text{'}}\times {\hslash }^{2}$$

According to equation 9, in order to estimate the non-parabolicity factor (*γ*), one need to have information about the effective mass of electron *m*_*e*_(*expt*.) of the QWs (need to be decoupled from the reduced mass, μ*), effective mass at the band edge (${m}_{e}^{\ast }$), wavenumber (${k}_{e}^{^{\prime} }$)/energy (${E}_{e}^{^{\prime} }$) corresponding to the non-parabolic conduction band.

**Table 2:** Increase in effective mass due to penetration of wave function into the barrier layer is estimated.

*l* (Å)	${\boldsymbol{\Delta }}{{\boldsymbol{m}}}_{{\boldsymbol{hh}}}^{{\boldsymbol{\ast }}}$ (penetration) (×*m*_0_)	${\boldsymbol{\Delta }}{{\boldsymbol{m}}}_{{\boldsymbol{e}}}^{{\boldsymbol{\ast }}}$ (penetration) (×*m*_0_)	${{\boldsymbol{m}}}_{{\boldsymbol{hh}}}^{{\boldsymbol{\ast }}}$(QW + penetration) (×*m*_0_)	*m*_*e*_(*expt*.) (×*m*_0_)	*E*_*g*_(*expt*.) (eV)	*E*_*hh*1_ (meV)	${{\boldsymbol{E}}}_{{\boldsymbol{e}}{\bf{1}}}^{^{\prime} }$ (meV)	*γ* (×10^−18^) (*m* ^2^)
190	0.0000	0.0000	0.350	0.169	1.5352	2.5	22.6	2.32
100	0.0000	0.0000	0.350	0.214	1.5665	7.9	50.0	1.18
50	0.0015	0.0001	0.352	0.303	1.6304	24.0	100.1	0.62
30	0.0060	0.0026	0.356	0.356	1.6926	48.0	139.5	0.48

Ground state energy and wavenumber of QW-electrons are estimated, and subsequently the non-parabolicity factor for the four QWs is summarized. Confinement energy for heavy hole (*E*_*hh*1_) is estimated by solving Schrödinger equation in FDM.

Heavy-hole valance band does not interact with conduction band and undergoes very small interaction with distant bands, which results in negligible non-parabolicity induced in heavy hole valence band^43, 48, 49^. Therefore the heavy hole effective mass of GaAs QW may be taken as *m*_*hh*_ = 0.35 *m*_0_ for this investigation^40, 50^. Light hole contribution is not taken into account because no light hole related feature is observed in PL spectra. Therefore, in our investigation, effective mass of electrons [*m*_*e*_(*expt*.), Table 2, Col. 6] is estimated by decoupling effective mass of heavy hole from the reduced effective mass of excitons (μ*, estimated through Magneto-PL experiment, Table 1) using following relation,

10 $$\frac{1}{{\mu }^{\ast }}=\frac{1}{{m}_{hh}^{\ast }}+\frac{1}{{m}_{e}(expt\mathrm{.)}}$$

Before the estimation of effective mass of electron, effect of penetration of heavy hole wave function into the barrier layer is also taken into account. Thereafter, transition energy of PL is used to estimate the confinement energy (${E}_{e1}^{^{\prime} }$) of electrons in a QW at the non-parabolic band, and the relation for the same is given below,

11 $${E}_{g}(expt\mathrm{.)}={E}_{g}(GaAs)+{E}_{e1}^{^{\prime} }+{E}_{hh1}-{E}_{b}$$

**Figure 5:**
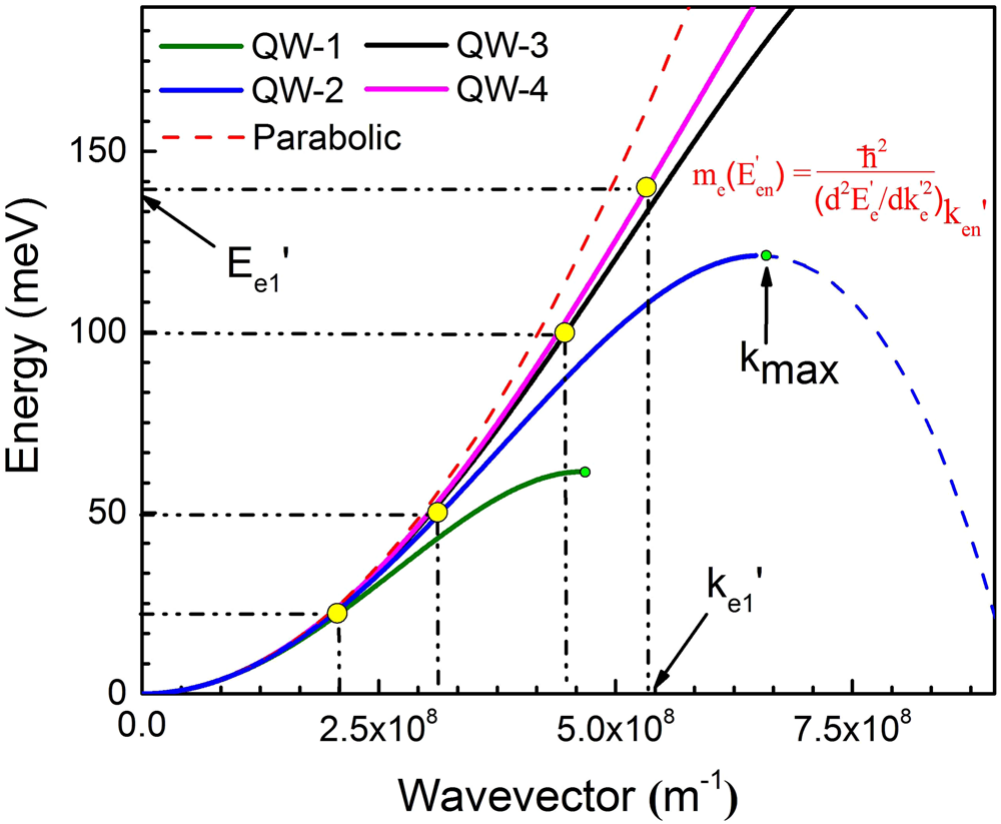
Dispersion curves of conduction band electron for the four QWs. Parabolic dispersion curve of bulk GaAs is also shown (red dashed line). It is clearly observed that the induced non-parabolicity in bands becomes dominant at higher ${k}_{e}^{^{\prime} }$ value. The yellow dot signs in dispersion curve indicates ground state (${E}_{e1}^{^{\prime} }$, ${k}_{e1}^{^{\prime} }$) of electron in QWs. Green dot sign signifies the maximum state up to which the dispersion curve is valid.

Here, *E*_*g*_(*expt*.) is the PL transition energy, *E*_*g*_(*GaAs*) = 1.519 eV is the bandgap of bulk GaAs at 4.2 K^34^, *E*_*hh*1_ is the confinement energy for heavy hole (estimated theoretically), *E*_*b*_ is the binding energy of the exciton (Table 1). Summary of ${E}_{e1}^{^{\prime} }$ for the QWs are shown in Table 2. Thereafter, equation 9 is used to estimate the non-parabolicity factor (*γ*) for the respective QW, with the help of *m*_*e*_(*expt*.), ${m}_{e}^{\ast }$ and ${E}_{e1}^{^{\prime} }$. Non-parabolic dispersion curves (E-k space) of electron for all the QWs are shown in Fig. 5. The variation of non-parabolicity factor and plot of non-parabolic dispersion curves with QW width are found to be important in our investigation, which can be compared with previous reported values of *γ* ^40, 43, 51, 52^. Note that the dispersion curves are valid in the range of 0 ≤ ${k}_{e}^{^{\prime} }$ ≤ *k*_*max*_, and for a quantum well with any realistic finite barrier ${k}_{e1}^{^{\prime} }$ is always less than *k*_*max*_^53^. Ground state confinement energy due to the non-parabolic dispersion relation for the QWs are shown in Fig. 5 (yellow dots). In order to re-extract the energy dependent effective mass of the QWs at a given state (${E}_{en}^{^{\prime} }$, ${k}_{en}^{^{\prime} }$) using the given dispersion curves (Fig. 5), the following relation may be used.

12 $${m}_{e}({E}_{en}^{^{\prime} })={[\frac{{\hslash }^{2}}{{d}^{2}{E}_{e}^{^{\prime} }/d{k}_{e}^{^{\prime} 2}}]}_{{k}_{en}}$$

Effective mass due to the non-parabolicity [${m}_{e}^{\ast }({E}_{en}^{^{\prime} })$] and wave function penetration into the barrier layer [${\rm{\Delta }}{m}_{e}^{\ast }(penetration)$] can be added to get the total effective mass of electrons [${m}_{e}^{\ast }(QW+peneration)$] for GaAs QWs as per the relation (4). As the dimension of a QW is reduced, ground state energy of electron shifts toward higher wavenumber/higher energy. It is found that the value of non-parabolicity factor (*γ*) decreases gradually with decrease in QW thickness, however the effect of non-parabolicity on the effective mass of the QWs becomes dominant at higher ${k}_{e}^{^{\prime} }$. Therefore, the estimated dispersion curves, shown in Fig. 5, well explain the higher effective mass of QWs, bulk effective mass at the band edge and also increase in confinement energy due to non-parabolicity of band.

### Quantification of atomic irregularity at the hetero-junction

**Figure 6:**
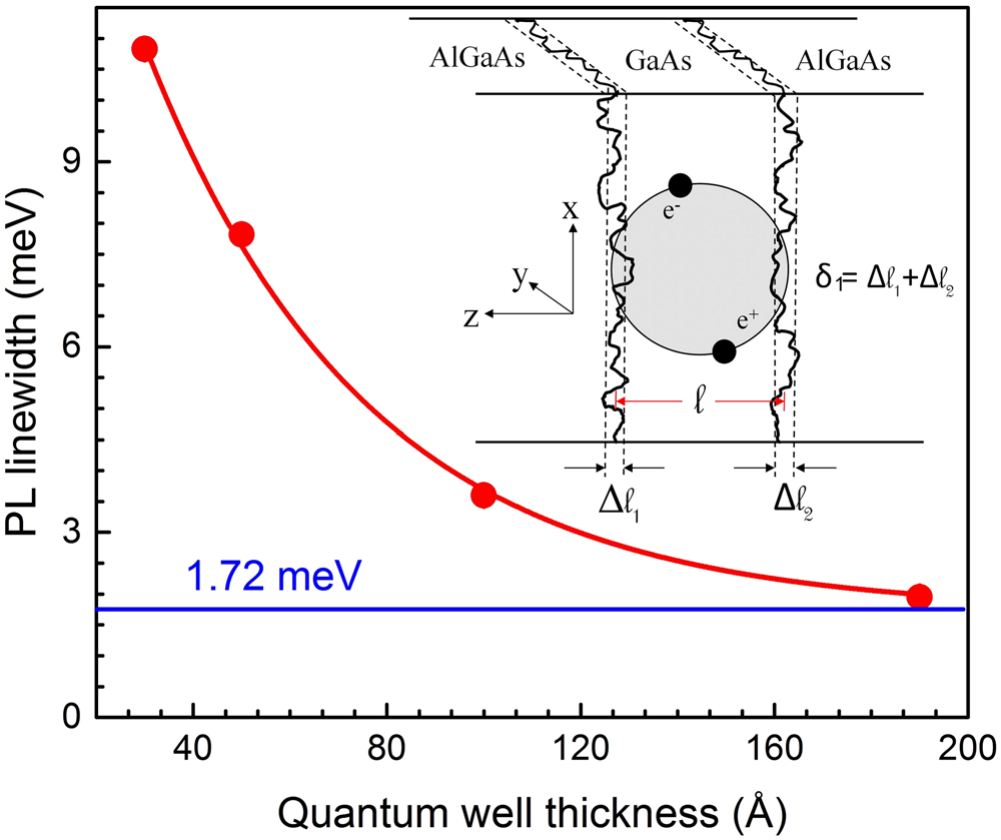
Variation of PL linewidth (*P*_1_) with QW thickness is shown. The minimum PL linewidth of AlGaAs/GaAs QWs is estimated to be (1.72 ± 0.05) meV. Inset of the figure shows, atomic irregularities at the hetero-interface resulting local variation in QW width. Excitons in narrow QWs substantially experience atomic irregularities at the hetero-junction, and undergo strong influence due to barrier layer.

Figure 4 shows that the spatial extent of electron/hole wave functions in QW-1 (*l* = 190 Å) is less than its QW width. Therefore, the charge carriers in QW-1 are not significantly influenced by the potential fluctuation caused by the atomic irregularities at the hetero-interface and barrier layer. On the other hand, for another 3 QWs spatial distribution of electron/hole wave function along the confinement direction is larger than the corresponding QW thickness (Fig. 4). Therefore, the increase in PL linewidth for a narrow QW is a consequence of penetration of wave function into the barrier layer with substantial experience of atomic irregularities at the hetero-junction. Monotonic decrease in PL linewidth (*P*_1_) with QW thickness saturates near (1.72 ± 0.05) meV [Fig. 6], which represents the minimum line-broadening corresponding to natural broadening (${\rm{\Delta }}{E}_{Natural}$), in-plane volume defects (${\rm{\Delta }}{E}_{Volume}$) and due to potential fluctuation (${\rm{\Delta }}{E}_{Pot\mathrm{.}fluct\mathrm{.}}$). Fraction of electron/hole wave function that penetrates into the barrier layer (*f*) is already computed numerically as shown in Fig. 4 and the values are summarized in Table 3. Thereafter, increase in PL linewidth due to the penetration of wave function into the barrier layer is estimated by,

13 $${\rm{\Delta }}{E}_{penetration}=\mathrm{(1}-f)\times {\rm{\Delta }}{E}_{A{l}_{0.3}G{a}_{0.7}As}$$

where, the PL linewidth of bulk Al_0.3_Ga_0.7_As layer is measured independently and found to be 16.6 meV. Finally, equation 1 is used to estimate the PL line broadening (${\rm{\Delta }}{E}_{Thickness}$) evolved due to the thickness fluctuation (*l* ± *δ*_1_) by decoupling the other terms. PL linewidth broadening due to this thickness fluctuation (${\rm{\Delta }}{E}_{Thickness}$) can be approximated by the energy expression of an infinite potential well as [$E(l-{\delta }_{1})-E(l+{\delta }_{1})$], where (*l* ± *δ*_1_) represents the effective thickness of the QW. Therefore, the amount of PL line-broadening acquired by the fluctuation in QW width can be expressed as follows,

14 $${\rm{\Delta }}{E}_{Thickness}=\frac{{\pi }^{2}{\hslash }^{2}}{{\mu }^{\ast }}[\frac{2l{\delta }_{1}}{{({l}^{2}-{\delta }_{1}^{2})}^{2}}]$$

The simple relation provided by equation 14 is good enough to estimate the values of *δ*_1_. Required μ* for these analysis is taken from the previously described Magneto-PL experiment. The *δ*_1_ estimated for the four QWs in our case varies from ±(0.14 − 1.6) monolayer (one monolayer = 2.83 Å) thickness of GaAs, which is also observed in TEM images. Similar value is also reported by other researchers from much more elaborated and complex measurements, such as cross sectional TEM, STM etc^54–57^. It can be understood that, out of plane defects get accumulated as the thickness of the QW increases. As a result higher number of defects are experienced by the excitons in a thick QW, and therefore contribute to the line-broadening of PL signal (Δ*E*), which is subsequently used to estimate fluctuation in the thickness of QWs (*δ*_1_). Therefore, a small influence of out of plane defects of the QWs may exist in the estimated value of *δ*_1_ for the QWs. Additionally, a fraction of QW thickness fluctuation (*δ*_1_) may average over the higher exciton Bohr radius in a thick QW, which can affect the PL linewidth. However, a small contribution of disorder averaging on the linewidth of PL spectra may depend on the quality of epilayer/hetero-interface, the kinetics of excitons and free exciton capture mechanisms^15, 58^. The above approach to investigate the atomic irregularity at the heterojunction and thickness fluctuation of a QW will be suitable for any set of QWs having different thickness and grown under identical conditions. However, the variation of composition and growth conditions among the QWs may lead to considerable error in the final result (*δ*_1_). This is because in such a situation the intentional/unintentional dopant density, disorder/defect density and the quality of hetero junction among the QWs may vary significantly. In view of this, contactless nature of PL technique becomes really attractive since it can provide a quantitative information on the inhomogeneities present in MQW sample, in a short time scale.

**Table 3:** Probability of finding excitons inside the barrier layer (*1*-*f* ) is computed by solving Schrödinger equation in FDM as shown in Fig. 4.

*f*	(*1*-*f* )	Δ*E*_*penetration*_ (meV)	${\boldsymbol{\Delta }}{{\boldsymbol{E}}}_{{\boldsymbol{Natural}}}^{{\bf{2}}}+{\boldsymbol{\Delta }}{{\boldsymbol{E}}}_{{\boldsymbol{Volume}}}^{{\bf{2}}}+{\boldsymbol{\Delta }}{{\boldsymbol{E}}}_{{\boldsymbol{Pot}}.{\boldsymbol{fluct}}.}^{{\bf{2}}}$ (meV)	PL linewidth (*P*_1_) (meV)	Δ*E*_*Thickness*_ (meV)	*δ*_1_ ± *Error* (Å)
0.9999	0.0001	0.00	1.72	1.95	0.91	±4.7 ± 0.4
0.9998	0.0020	0.03	1.72	3.64	3.21	±2.2 ± 0.3
0.9799	0.0201	0.33	1.72	7.82	7.62	±1.1 ± 0.2
0.9132	0.0868	1.44	1.72	10.83	10.56	±0.4 ± 0.2

Spectral broadening due to wave function penetration is estimated and thereafter PL linewidth broadening due to the thickness fluctuation are estimated from equation 1. Summary of *δ*_1_ estimated for all the QWs are shown.

### Magnetic field effects on the free and bound exciton luminescence

**Figure 7:**
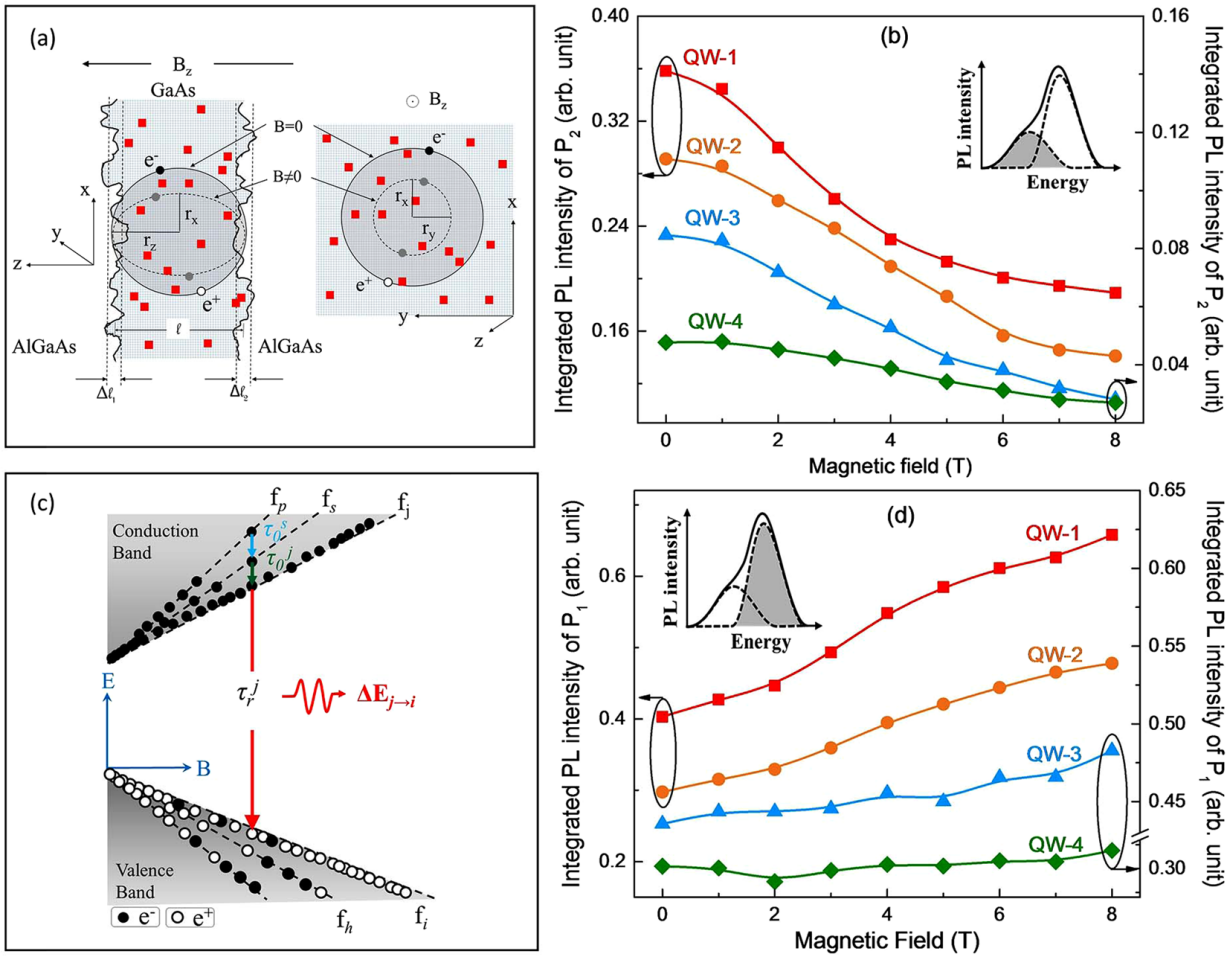
(**a**) Spatial extent of exciton inside the QW is shown. Red dots in the figure represent random defects, disorder, and impurity (*δ*_2_) in GaAs layer. In presence of magnetic field, spatial extent of exciton reduces and hence experience less volume-defects (*δ*_2_). However, experience of hetero-interface potential fluctuation (*δ*_1_) remains nearly invariant. (**b**) Figure shows the trend of decrease in *P*_2_ with magnetic field follows the similar trend as magnetic field driven confinement of exciton. (**c**) Shows the excitonic radiative recombination between *j* ^*th*^ state of conduction band and *i* ^*th*^ state of valence band (lifetime ${\tau }_{r}^{j}$). Increase in magnetic field results in splitting of energy levels (i.e. Landau levels). Carrier density (*n*_*j*_) in each Landau level may significantly change with increase in magnetic field. The symbol ${\tau }_{0}^{k}$ signifies average relaxation lifetime of charge carriers to the energy level *k*. (**d**) Integrated intensity of Gaussian function peak *P*_1_, originated from free exciton radiative recombination, increases with magnetic field. Data is normalized by the excitation power density received by the respective QW in MQW sample.

A careful observation on the Magneto-PL spectra [Figs 1, 2 and 3] indicate that the asymmetry in PL line-shape is nearly disappeared at 8 T. Added to this, a simultaneous increase in PL intensity is also observed with magnetic field. In order to understand these observations, the kinetics of spherical/ellipsoidal excitons in QWs under magnetic field are carefully investigated. Magnetic field along the growth direction of the QW (*B*_*z*_) results in in-plane (x-y) confinement of exciton. Consequently, the confined excitons in a QW experience less number of random defects/disorder (*δ*_2_) that are embedded in the QW layer, as schematically shown in Fig. 7(a). However, it can be understood that the atomic irregularities (*δ*_1_) experienced by excitons at the hetero-junction depends only on the dimension (*l*) of the QW, and may not be much influenced by the application of magnetic field *B*_*z*_ [Fig. 7(a)]. As a result of this magnetic field driven confinement of exciton, it can be expected that the PL line-shape should also be affected by the decreased radiative-recombination from the defect states *δ*_2_ (volume defects). In the classical regime (B < *B*_*c*_), charge carriers produce cyclotronic motion, with radius (r) inversely proportional to *B*. However, at sufficiently higher magnetic field (quantum regime, B ≥ *B*_*c*_), radius varies as inversely proportional to the square root of *B* ^31^.

15 $${r}_{x}={r}_{y}=\frac{{\mu }^{\ast }{v}^{2}}{eB};B < {B}_{c}$$

16 $${r}_{x}={r}_{y}={\mathrm{(2}N+1)}^{\frac{1}{2}}{\xi }_{B}={\mathrm{(2}N+\mathrm{1)}}^{\frac{1}{2}}\sqrt{\frac{\hslash }{eB}};B\ge {B}_{c}$$

where, *v* is the velocity of charge carrier, *N* represents the index of Landau level. Therefore, at low magnetic field the spatial volume enclosed by an exciton [${V}_{xyz}=\mathrm{(4/3)}\times \pi \times {r}_{x}{r}_{y}{r}_{z}$] reduces as inversely proportional to the square of *B*, and at relatively higher magnetic field *V*_*xyz*_ reduces as inversely proportional to *B*. Integrated PL intensity of bound exciton peak *P*_2_ (responsible for asymmetry in PL) of QWs decay with magnetic field, and this observed decrease of *P*_2_ follow the similar trend as magnetic field driven decrease in spatial extent (volume) of excitons, which is clear in Fig. 7(b). Therefore, it can be concluded that the disorder-defects present in GaAs layer itself is the origin of peak *P*_2_, which is suppressed due to magnetic field driven confinement of exciton in smaller region of space. It is to be noted that the spatial extent of excitons in QW depends on the thickness of the QW, and is observed that at zero magnetic field excitons confined in very narrow QW are ellipsoid in shape^16, 59^. To some extent, this is also the case for the excitons in our thinnest QW (30 Å). Under such situation, PL line-broadening may be reduced due to the decreased influence of ${\rm{\Delta }}{E}_{Thickness}$ in high magnetic field^16, 36^. In addition to this, effect of magnetic field on the optical properties of exciton may also depend on the lateral extent of inhomogeniety at the hetero-interface. In our case, atomic irregularity at the hetero-junction is nearly uniform and within 1–2 monolayer thickness (conclusion from the TEM image); and throughout the hetero-interface there are no such terrace/convex shaped interface with dimension comparable to spatial extent of excitons are observed, which was observed earlier^16, 59^.

Further, the magnetic field driven confinement of exciton results in greater overlap of electron/hole wave function in the QWs, which may lead to greater oscillator strength of exciton i.e. higher PL intensity. The influence of magnetic field on the PL intensity of exciton can be realized by the rate equation, considering the relaxation and recombination of charge carrier among the Landau levels [Fig. 7(c)]. Non-radiative relaxation lifetime and radiative recombination lifetime are denoted as ${\tau }_{0}^{s}$ and ${\tau }_{r}^{s}$, respectively [Fig. 7(c)]. The symbol *S* stands for the index of the corresponding energy level. In presence of sufficient empty states at lower energy level *f*_*i*_, the rate of radiative recombination probability (PL intensity) between the energy states *f*_*i*_ and *f*_*j*_ can be expressed as follows^22, 60, 61^,

17 $${R}_{j\to i}=-\frac{d{n}_{j}}{dt}=\frac{{n}_{j}}{{\tau }_{r}^{j}}-a\frac{{n}_{s}}{{\tau }_{0}^{j}}+b\sum _{k\ne j}\,\frac{{n}_{k}}{{\tau }_{0}^{s}}$$

Here, *n*_*j*_, *n*_*s*_ are the population of charge carriers at the energy level *f*_*j*_ and *f*_*s*_, respectively. The symbols *a* and *b* are in general constant for a QW, and can be a function magnetic field. Negative sign in equation 17 signifies that in absence of electron-hole pair generation, the rate of radiative recombination decreases with time. Second term of equation 17 represents the non-radiative relaxation of charge carrier from *f*_*s*_ state to *f*_*j*_, with average relaxation lifetime ${\tau }_{0}^{j}$. The last term signifies the relaxation of charge carriers to the energy level other than *f*_*j*_, which do not contribute to our concerned recombination between *f*_*j*_ to *f*_*i*_ state. Note that with increase in magnetic field, the degeneracy of Landau levels increases and also the separation between the energy levels changes. Therefore, the population of charge carrier in the Landau energy levels (*n*_*j*_), and the relaxation (*τ*_0_)/recombination lifetime (*τ*_*r*_) of charge carrier may be significantly changed under the magnetic field, which leads to a dynamical steady state in the system^62^. The above rate equation (Eq. 17) readily shows that radiative recombination probability (PL intensity) increases with decrease in effective lifetime ($1/{\tau }_{effective}=1/{\tau }_{r}+1/{\tau }_{0}$) of charge carriers and with increase in carrier density (*n*_*j*_). Therefore, the experimentally observed increase in integrated PL intensity of *P*_1_ with magnetic field [Fig. 7(d)] might be the effect of decreased effective lifetime, and due to the accumulation of charge carrier at the lower energy levels *f*_*i*_ and *f*_*j*_ [Fig. 7(c)]. A similar phenomenon of increase in PL intensity with decrease in lifetime of charge carriers of QW is experimentally observed by Harrison *et al*.^36^. This decrease in radiative recombination lifetime could be due to magnetic field driven confinement of excitons in smaller region of space (x-y plane), which results in grater overlap of electron and hole wave functions i.e. Coulomb interaction between two oppositely charged particles (electron/hole) becomes stronger. An analytical solutions of equation 17, considering five discrete energy levels, is already shown by F. Adler *et al*.^22^. It is to be noted that in case of very thin QWs (e.g. QW-4), significant portion of wave function is penetrated into the barrier layer and the PL line-shape gets considerably broadened due to the large experience of atomic irregularities at the hetero-interface (fluctuation in quantum confinement of charge carrier). In addition to this, excitons are already in smaller spatial dimension and possess higher exciton binding energy (*E*_*b*_). These could be the possible reason for the observed gradual effects of magnetic field driven in-plane confinement of exciton on the PL line-shape of narrow QWs [Fig. 7(b,d)].

## Conclusions

In summary, we have investigated on the effects of charge carrier confinement on the effective mass of excitons, origin of PL line-shape and the effects of magnetic field on the free and bound exciton luminescence, using the Magneto-PL spectroscopy. It is concluded that the increase in effective mass at low QW thickness is a consequence of wave function penetration into the barrier layer with significant effect of non-parabolicity in conduction band. In addition to this, the atomic irregularities at the hetero-junction of AlGaAs/GaAs QWs are quantitatively estimated by modeling the PL linewidth of various QWs of different thicknesses that are grown under identical conditions. Estimated values of atomic irregularities at the Al_*x*_Ga_1−*x*_As/GaAs hetero-interface (*δ*_1_) are close to one monolayer thickness of GaAs layer, which shows the superior quality of grown AlGaAs/GaAs interface. The simple model constructed here may not provide exact quantitative results of *δ*_1_ for the QWs grown under different growth conditions. Therefore, the MQW system with varying QW thickness, but by keeping the same background parameters is found to be a key recipe of our work. Further, variation of PL line-shape with magnetic field helped in realizing the effect of magnetic field on the suppression of bound exciton and enhancement of free exciton luminescence at different dynamical steady state. Increase in excitonic radiative recombination efficiency with simultaneous decrease in disorder (*δ*_2_) related recombination are found to be the major effects of magnetic field. Such effects can be highly useful in opto-electronics devices for the enhancement of optical efficiency with reduced frequency band-width output.
